# Short-term Internet search using makes people rely on search engines when facing unknown issues

**DOI:** 10.1371/journal.pone.0176325

**Published:** 2017-04-25

**Authors:** Yifan Wang, Lingdan Wu, Liang Luo, Yifen Zhang, Guangheng Dong

**Affiliations:** 1 Department of Psychology, Institute of Psychological and Brain Sciences, Zhejiang Normal University, Jinhua, P.R. China; 2 Department of Psychology, University of Konstanz, Konstanz, Germany; 3 State Key Laboratory of Cognitive Neuroscience and Learning and IDG/McGovern Institute for Brain Research, Beijing Normal University Beijing, China; 4 Institute of Psychological and Brain Sciences, Zhejiang Normal University, Jinhua, P.R. China; Hangzhou Normal University, CHINA

## Abstract

The Internet search engines, which have powerful search/sort functions and ease of use features, have become an indispensable tool for many individuals. The current study is to test whether the short-term Internet search training can make people more dependent on it. Thirty-one subjects out of forty subjects completed the search training study which included a pre-test, a six-day’s training of Internet search, and a post-test. During the pre- and post- tests, subjects were asked to search online the answers to 40 unusual questions, remember the answers and recall them in the scanner. Un-learned questions were randomly presented at the recalling stage in order to elicited search impulse. Comparing to the pre-test, subjects in the post-test reported higher impulse to use search engines to answer un-learned questions. Consistently, subjects showed higher brain activations in dorsolateral prefrontal cortex and anterior cingulate cortex in the post-test than in the pre-test. In addition, there were significant positive correlations self-reported search impulse and brain responses in the frontal areas. The results suggest that a simple six-day’s Internet search training can make people dependent on the search tools when facing unknown issues. People are easily dependent on the Internet search engines.

## Introduction

Technology has been a vital part of our everyday life. It changes the ways we live, learn, work and play. The human brain, as the most sensitive organs, is under change in response to the modern world. It is developing and adapting to outside stimuli [1]. The Internet search engines have become an indispensable tool for many individuals. It is also regarded as one of the most important inventions in the past few decades. Specifically, the using of search engines has changed ways we finding and storing information by making much information readily available as “external memory source” [2].

The influence of Internet search has attracted attention around the world. Scholars have postulated that the popularity of Internet search may lead individuals to lose the ability to process and store information effectively [3]. Also, a few studies tried to find the potential neural mechanisms underlying searching behaviors. For example, Small et al. found that a short-term (5 days) Internet search training can change neural circuits involved in decision-making and complex reasoning in aged adults [4]. A research also found decreased brain activities in the middle frontal gyrus and temporal gyrus during recollection process after a six-day Internet search practice [5]. Searching for information online made people mistakenly believe that they have more knowledge as indexed by an increase in self-assessed knowledge [6]. People using Internet search as tools to find and remember information showed lower brain activations in declarative-memory-related brain regions, and recent Internet search using may promote motivations to use it [7].

Given these data, it is interesting for us to investigate whether using search engine would make people become more dependent on it as it is so ease of use and powerful in providing all kinds of information. Further, what are the neural mechanisms underlying this process? Studies have found that the “Google generation” (people who born after 1993) demonstrated less confidence in their answers than older people [8], which might be caused by their dependence on the search tools. Also, people became better at remembering where information was stored than remembering the information itself, caused by Internet search using [2]. Despite of the importance of such cross-sectional studies, they are limited with respect to investigating possible “cause and effect” influences. Thus, longitudinal studies might help inform how Internet search using may alter behaviors and brain functions. Internet search using might affect the habitual thinking when facing new information. Thus, this study sought to explore the possible effects of short-term Internet-search training on people’s behaviors when facing unknown issues.

The current study designed a longitudinal study to investigate whether the short-term Internet search-training can made people more dependent on it by comparing people’s behavioral responses and brain activities during the pre- and post- tests. According to the empirical studies we stated above, we expected that the Internet search using would make people dependent on it. At the behavioral level, it was also expected that people might show a higher level of impulse to search the webs when encountering unknown items. In addition, at the neurological level, we hypothesized that the impulse and impulse control related brain regions, such as dorsolateral prefrontal cortex (DLPFC) [9–12], and anterior cingulate cortex (ACC) (See a review [13]) would show higher activations when facing unknown items after the 6 days internet search training.

## Methods and materials

The experiment conforms to The Code of Ethics of the World Medical Association (Declaration of Helsinki). The Human Investigations Committee of Zhejiang Normal University approved this research. Forty university students were recruited through advertisements. 31 subjects (14 females, 17 males; Mean age = 20.5 ±1.1 years) completed the whole study. All Subjects were free of psychiatric disorders (including major depression, anxiety disorders, schizophrenia, and substance dependence disorders) as assessed by the MINI. All subjects were measured by an Internet searching using questionnaire [14], which showed that all subjects used the Internet search regularly for such purposes.

### Experiment procedures

**Fig 1:**
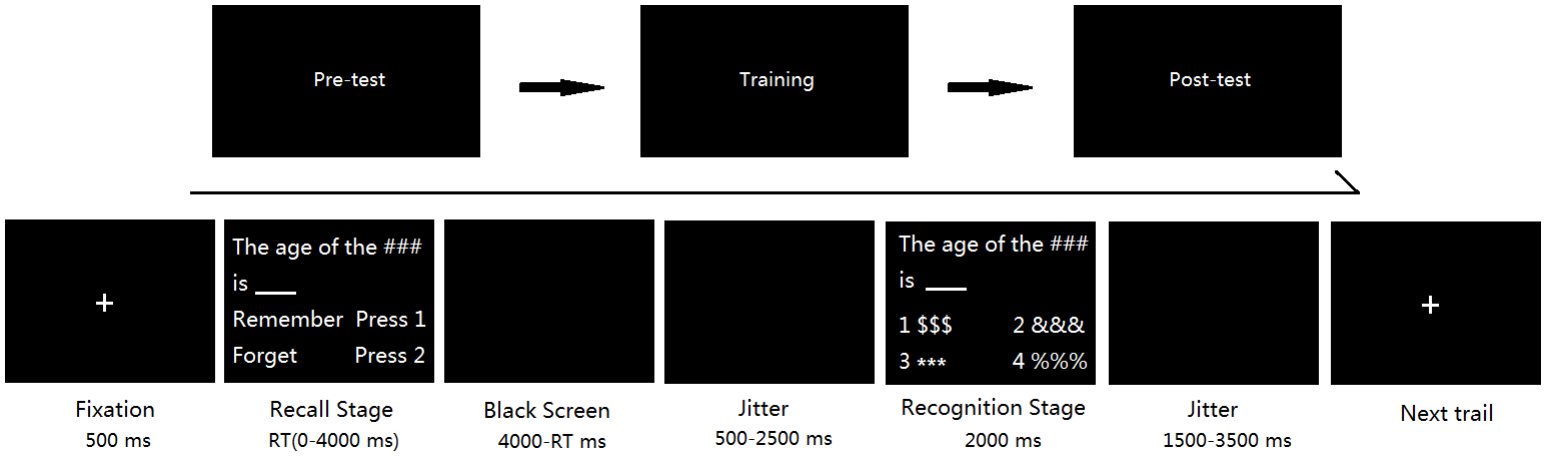
The timeline of the “recall and recognition” task.

The experiment consisted of three steps: pre-test, six days of training, and post-test. During the pre-test and post-test, subjects were instructed to perform a ‘search-remember-recall and recognition’ task and then their brain activities were recorded in the fMRI scanner (Fig 1).

The tasks in pre- and post-training are of the same type but differ in contexts, which is to avoid possible ‘repetition effects’. We designed two copies of the task with different items (Copy A, B). Half of the Subjects participated in an “A-B” sequence, and the other half received a “B-A” sequence in their pre- and post-training scans.

### Task

The task was described in our previous study [15,16] and as follows.

Subjects were asked to search for answers to forty questions by using an Internet search engine and to remember those answers within one hour before scanning. Taking notes were forbidden, which in order to promote memory generation and recall processes. Subjects were asked to perform a 5-minute distraction task (continuously subtract 4 from 99) and complete several questionnaires (taking at least 5 minutes), which is in order to avoid subjects’ recitation during the waiting period after the search-remember process.

During fMRI, subjects were asked to perform a ‘recall and recognition’ task. In each trial of the task, a fixation was presented for 500ms, and then came the recall stage, which lasted up to 4000ms. At the recall stage, one of the forty questions was presented and subjects were asked to answer ‘remember’ or ‘forget’ by pressing relevant button. The question was presented for 4000 ms and then the screen turned black after the button pressing. The black screen lasted for 500 to 2500ms. The following is a recognition stage. At this stage, Subjects were asked to choose an answer for the question from an answer list. This stage lasted for up to 2000ms (terminated by a button press), which was followed by a black screen with a jittered interval ranging from 1500ms to 3500ms. Stimuli were presented and behavioral data were collected using E-prime software (Psychology Software Tools, Inc.). We focused analyses on the recall stage in the current study.

### The impulse trials

During the recollection process in the fMRI scan, besides these 40 remembered questions, some new questions that they had never learned before would appear randomly, which was intended to elicit their impulses to use the relevant search tools. 10 novel questions were randomly presented. None of them was presented in the first 5 trials. These novel items were both presented in pre- and post- tests and used as odd stimuli with small probability. The current study focused on the behavioral and brain responses between pre- and post-tests when people encountered these novel questions of low probability.

All of the questions are related to uncommon topics (for example, the age of the first animal that people sent to space), which is to avoid potential effects of subjects’ previous knowledge. Besides this, during the searching-remembering period, they were asked to identify questions to which they already knew the answers. We would exclude these items from further analysis.

Subjects were told that they would receive 50 Yuan (≈8 $) for participating this study, and an additional 0–40 Yuan if they could respond accurately, which is to motivate their response correctly. Specifically, if they responded ‘remember’ in the ‘recall’ stage and chose the correct answer in the ‘recognition’ stage, they would gain 1 Yuan for each trial. If they responded ‘remember’ in the ‘recall’ stage and chose the incorrect answer in the ‘recognition’ stage, they would lose 1 Yuan. The other responses would be neither rewarded nor punished.

### The ‘training’ process

The training lasted for 6 consecutive days. During the training stage, subjects were ‘trained’ on Internet search for more than one hour per day. In training, subjects were asked to finish six search tasks in a random order over the 6 days’ training, each task consisted of 80 fill-in-the-blank questions. Subjects were asked to seek for answers via Internet search engine. To increase their motivations to search for the best answers, subjects were paid up to 20 Chinese Yuan per day for their participation (20 * accuracy rates (%)).

### Behavioral measures

A short self-reported questionnaire was presented to subjects after the fMRI scan. The questionnaire measured their subjective experiences including strength of impulses for Internet search when facing un-learned questions [17].

### fMRI data collection and pre-processing

Detailed parameters about data collection were described in previously published paper [5]. In current study, a general linear model (GLM) was applied to identify BOLD activation in relation to brain activities. Different types of trials were separately convolved with a canonical hemodynamic response function to form task regressors. The duration of each trial is 4000ms. The GLMs included a constant term per run. Six head-movement parameters derived from the realignment stage were included to exclude motion-related variances (subjects will be excluded from further analysis if they exceeded movement criteria of 2mm or 2 degrees between TRs). A GLM approach was used to identify voxels that were significantly activated for each event during recall stage was modeled.

### Pre-post comparisons

We compared the brain responses in those novel trials with a paired-sample *t*-test (post-test > pre -test). Family-wise error (FWE) thresholds were determined using AlphaSim. Significant clusters (FWE-corrected, *P* < 0.01) at *P* < 0.01, two-tailed, uncorrected, with an extent of at least 45 voxels, based on the unresliced voxel size (3*3*3). All these steps were performed using Neuroelf (http://neuroelf.net). The smoothing kernel used during simulating false-positive (noise) maps using AlphaSim was 6mm, and was estimated from the residual fields of the contrast maps being entered into the one-sample *t*-test.

### Correlation analyses between behavioral and brain performances

We first compared the brain activation between pre- and post-tests and then took the surviving clusters as ROIs in further analyses. A representative BOLD beta value was obtained by averaging the signal of all the voxels within the ROI. We performed the correlations between brain activations changes between pre- and post- tests in frontal areas and self-reported searching impulse changes when facing new items.

## Results

### Behavioral performance

In self-reported responses, subjects reported higher impulse to use search engines when facing novel trials in post-test (4.48±1.235) than in pre-test (3.66±1.383) (*t (30)* = 4.590, *p* = 0.000, *d* = 1.68).

### Imaging results

**Fig 2:**
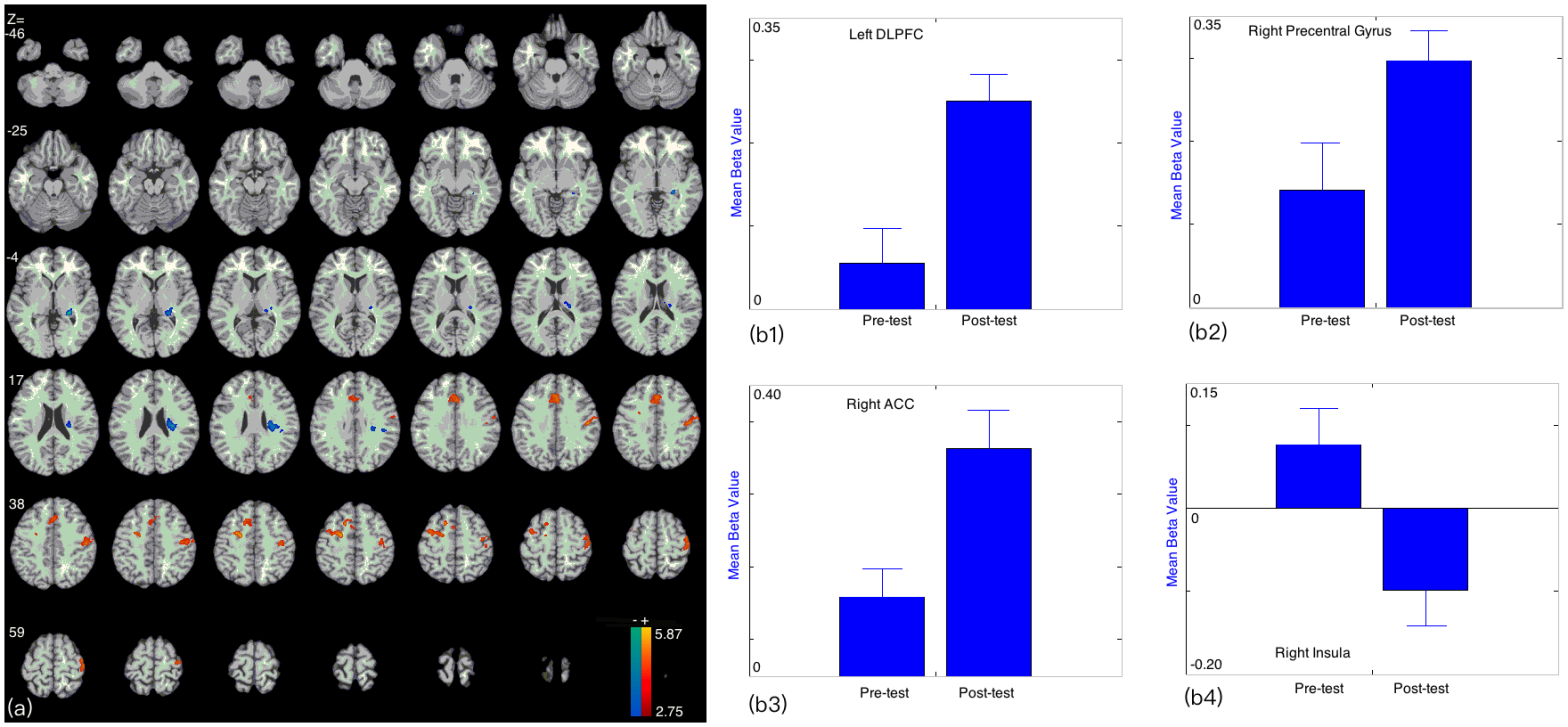
(a) Brain areas showing different activations when comparing the post-test to pre-test; (b1, b2, b3, b4) Beta figures of the survived clusters in pre- and post-tests.

When comparing to pre-test, the post-test are associated with higher brain activations in left DLPFC, right precentral gyrus (which also stretched to DLPFC), and right ACC (Fig 2a); The beta figures showed that these differences were caused by the increased brain activations in post-test (Fig 2b1, 2b2 and 2b3). In addition, lower brain activations were found in right insula, and the difference was caused by the decreased brain activations in post-test (Fig 2a and 2b4, Table 1).

**Table 1:** Regional brain activity changes in recall stage in post-test minus pre-test in facing the new unlearned trials.

Cluster Number	x,y,z ^a^	Peak Intensity	Cluster Size ^b^	Region ^c^	Brodmann’s Area
**1**	21, -3, 48	4.948	80	L Dorsolateral Prefrontal Cortex	6, 9
**2**	3, 18, 33	4.079	158	R Anterior Cingulate Cortex	32
**3**	36, -15, 66	4.010	186	R Precentral Gyrus	4,6
**4**	30, -21, 24	-4.083	112	R Insula	13

^a^ Peak MNI Coordinates.

^b^ Number of voxels. We first identified clusters of contiguously significant voxels at an uncorrected threshold *p*<0.01, as also used for display purposes in the figures. We then tested these clusters for cluster-level FWE correction *p*<0.01 and the AlphaSim estimation indicated that clusters with 60 contiguous voxels would achieve an effective FWE threshold p<0.01. Voxel size = 3*3*3.

^c^ The brain regions were referenced to the software Xjview (http://www.alivelearn.net/xjview8) and verified through comparisons with a brain atlas.

### Correlation results

**Fig 3:**
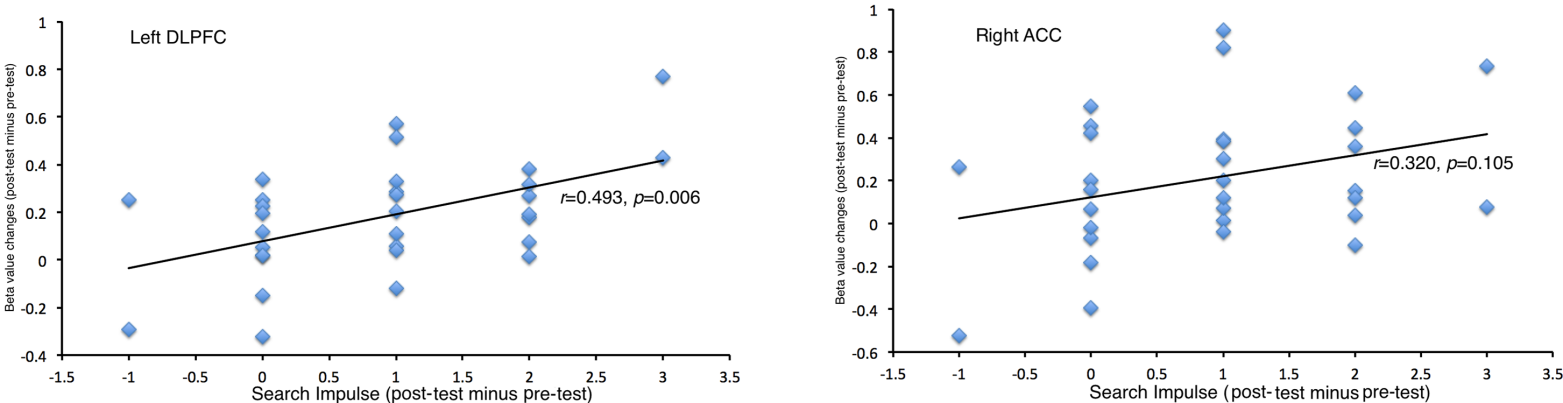
Correlations between brain activation changes and self-reported impulse changes to unknown trials between post- and pre- tests in (a) left DLPFC, and (b) right ACC.

Positive correlations were found between the brain activation changes (post-pre) in left DLPFC (*r* = 0.493, *p* = 0.006) and right ACC (*r* = 0.320, *p* = 0.105) activation and the changes (post-pre) in self-reported impulse to use Internet search (Fig 3a and 3b).

## Discussion

This study investigated behavioral and brain responses to unknown items in pre- and post-tests. As expected, the results showed that impulse control related brain regions, such as the DLPFC and ACC, were found to be more activated in post-test than in pre-test when facing unknown questions. The DLPFC was proved responsible for impulse control in controlling social behaviors [9], healthy choices [11], body weight [18], and decision making [19,20], etc. Its role can be found in most of the situations that require impulse control. In addition, the higher DLPFC activation usually suggests more endeavors engaged in impulse control [9,18–20]. The ACC is also one of the most heavily studied regions of the brain, which has been proved to be involved in the resistant to distraction or interference [21–24]. It plays a critical role in regulating [22,23] and monitoring behaviors [21,24]. The higher ACC activation usually suggests more endeavors engaged in the controlling process [18,23].

In current study, the enhanced brain activations in ACC and DLPFC in post-test suggest that more endeavors were engaged in controlling their impulse when facing new trials. The higher executive control during this process can be used as an index of higher impulse elicited in this situation; it suggests that the post-test elicited more search impulse than pre-test when facing unknown questions. The beta figures of these survived clusters showed that the difference was caused by the enhanced brain activations in post-test. Subjects reported stronger impulses to search the Internet in post-test comparing to pre-test, which were consistent with imaging results and suggested that Internet use training increased the impulse to use the Internet. The positive correlation between DLPFC activation and self-reported impulses to search the Internet suggests that the DLPFC activation may also link to Internet-use motivations as it does in drug craving in addictions [25,26].

The behavioral and imaging results in current study raised the possibility that the recent Internet search using promoted motivations to use the Internet and this may lead to a reliance on search tools when facing new situations. This explanation was supported by a previous study, which found that epistemic curiosity enhances the activities of motivation and memory system[27]. So, novel stimuli triggered different levels of curiosity and then the curiosity facilitated the behavior of Internet search. Inconsistent with the interpretation, Fisher’s research reported people believed that their brains worked “harder” after searching for information through the Internet [28]. However, our results suggest that online search does affect patterns of neural activity, but it doesn't necessarily make people think “harder”. From another perspective, the results also suggest that the 6-day, one-hour-per-day’s Internet search using made people more dependent on this tool. Taken together, the findings indicate that short-term Internet searching appears to promote motivations to use the Internet.

Another brain region survived after correction located in the insula, the post-test showed lower insula activation than pre-test. The insula is considered as a limbic-related cortex, which plays a role in awareness [29,30], body representation [31], and subjective emotional experience [32,33], etc. Our data cannot provide a reasonable explanation for its involvement and role in searching behaviors. Further studies are warranted to further investigate its role during this process.

The brain is the source of behavior, but in turn it is modified by the behaviors it produces [34]. The modern technologies are really attractive because of their powerful functions, friendly interface, ease of use and reliability [1,35]. The Internet search engine has made information extremely accessible; we can find what we want by simply typing some keywords. As studies suggested that people may use Internet search engines as ‘external memory drives’ in a manner that may diminish the importance of using brain-based memories [2]. It has lowered our burden to remember things. We are exposed to large amounts of information at every moment and cannot convert all short-term memory into long-term memory. However, by virtue of Internet search, we may achieve the required resources instantaneously rather than remembering all things. Psychologically, it allows us to better adapt to the complex and changeable environment. In all, people might be easily ‘addicted’ to this new types of technologies. All of these features of the Internet search might be the reason for why the short-term Internet search training makes people more dependent on search engines when facing unknown questions.

## References

[1] Loh KK, Kanai R (2015) How Has the Internet Reshaped Human Cognition? Neuroscientist. 10.1177/1073858415595005 26170005

[2] Sparrow B, Liu J, Wegner DM (2011) Google Effects on Memory: Cognitive Consequences of Having Information at Our Fingertips. Science 333: 776–778. 10.1126/science.1207745 21764755

[3] Carr N (2010) Is Google making you stupid? The Times Saturday Review 14: 1–2.

[4] Small GW, Moody TD, Siddarth P, Bookheimer SY (2009) Your brain on Google: patterns of cerebral activation during internet searching. Am J Geriatr Psychiatry 17: 116–126. 10.1097/JGP.0b013e3181953a02 19155745

[5] Dong G, Potenza MN (2016) Short-term Internet-search practicing modulates brain activity during recollection. Neuroscience 335: 82–90. 10.1016/j.neuroscience.2016.08.028 27555549

[6] Fisher M, Goddu MK, Keil FC (2015) Searching for Explanations: How the Internet Inflates Estimates of Internal Knowledge. Journal of Experimental Psychology-General 144: 674–687. 10.1037/xge0000070 25822461

[7] Dong G, Potenza MN (2015) Behavioral and brain responses related to Internet search using on memory. European Journal of Neuroscience: n/a–n/a. 10.1111/ejn.13039 26262779

[8] Nicholas D, Rowlands I, Clark D, Williams P (2011) Google Generation II: web behaviour experiments with the BBC. Aslib Proceedings 63: 28–45.

[9] Steinbeis N, Bernhardt BC, Singer T (2012) Impulse control and underlying functions of the left DLPFC mediate age-related and age-independent individual differences in strategic social behavior. Neuron 73: 1040–1051. 10.1016/j.neuron.2011.12.027 22405212

[10] Miller EK, Cohen JD (2001) An integrative theory of prefrontal cortex function. Annu Rev Neurosci 24: 167–202. 10.1146/annurev.neuro.24.1.167 11283309

[11] Hare TA, Camerer CF, Rangel A (2009) Self-control in decision-making involves modulation of the vmPFC valuation system. Science 324: 646–648. 10.1126/science.1168450 19407204

[12] Dong G, Wang L, Du x, Potenza M (in press) Gaming increases craving to gaming-related stimuli in individuals with Internet gaming disorder. Biological Psychiatry: CNNI 2. 10.1016/j.bpsc.2017.01.002 29560926

[13] Shenhav A, Botvinick MM, Cohen JD (2013) The expected value of control: an integrative theory of anterior cingulate cortex function. Neuron 79: 217–240. 10.1016/j.neuron.2013.07.007 23889930 PMC3767969

[14] Wang Y, Wu L, Zhou H, Xu J, Dong G (2016) Development and Validation of a Self-reported Questionnaire for Measuring Internet Search Dependence. Front Public Health 4: 274 10.3389/fpubh.2016.00274 28066753 PMC5167696

[15] Dong G, Potenza M (2015) Behavioural and brain responses related to Internet search and memory. Eur J Neurosci 42: 2546–2554. 10.1111/ejn.13039 26262779

[16] Dong GH, Wang YF, Potenza MN (2016) The activation of the caudate is associated with correct recollections in a reward-based recollection task. Human Brain Mapping 37: 3999–4005. 10.1002/hbm.23290 27329532 PMC6867516

[17] Wang Y, Wu L, Zhou H, Xu J, Dong G (2016) Development and Validation of a Self-reported Questionnaire for Measuring Internet Search Dependence. Front Public Health 4. 10.3389/fpubh.2016.00274 PMC5167696 28066753

[18] Kishinevsky FI, Cox JE, Murdaugh DL, Stoeckel LE, Cook EW 3rd, et al (2012) fMRI reactivity on a delay discounting task predicts weight gain in obese women. Appetite 58: 582–592. 10.1016/j.appet.2011.11.029 22166676

[19] Kable JW, Glimcher PW (2007) The neural correlates of subjective value during intertemporal choice. Nat Neurosci 10: 1625–1633. 10.1038/nn2007 17982449 PMC2845395

[20] McClure SM, Laibson DI, Loewenstein G, Cohen JD (2004) Separate neural systems value immediate and delayed monetary rewards. Science 306: 503–507. 10.1126/science.1100907 15486304

[21] Botvinick M, Nystrom LE, Fissell K, Carter CS, Cohen JD (1999) Conflict monitoring versus selection-for-action in anterior cingulate cortex. Nature 402: 179–181. 10.1038/46035 10647008

[22] Botvinick MM, Cohen JD (2014) The computational and neural basis of cognitive control: charted territory and new frontiers. Cogn Sci 38: 1249–1285. 10.1111/cogs.12126 25079472

[23] Cohen JD, Dunbar K, McClelland JL (1990) On the control of automatic processes: a parallel distributed processing account of the Stroop effect. Psychol Rev 97: 332–361. 2200075 10.1037/0033-295x.97.3.332

[24] Botvinick MM, Cohen JD, Carter CS (2004) Conflict monitoring and anterior cingulate cortex: an update. Trends Cogn Sci 8: 539–546. 10.1016/j.tics.2004.10.003 15556023

[25] Potenza MN, Hong KI, Lacadie CM, Fulbright RK, Tuit KL, et al (2012) Neural correlates of stress-induced and cue-induced drug craving: influences of sex and cocaine dependence. Am J Psychiatry 169: 406–414. 10.1176/appi.ajp.2011.11020289 22294257 PMC3690485

[26] Kober H, Lacadie CM, Wexler BE, Malison RT, Sinha R, et al (2015) Brain Activity During Cocaine Craving and Gambling Urges: An fMRI Study. Neuropsychopharmacology. 10.1038/npp.2015.193 PMC5130138 26119472

[27] Kang MJ, Ming H, Krajbich IM, Loewenstein G, Mcclure SM, et al (2009) The Wick in the Candle of Learning: Epistemic Curiosity Activates Reward Circuitry and Enhances Memory. Psychological Science 20: 963–973. 10.1111/j.1467-9280.2009.02402.x 19619181

[28] Fisher M, Goddu MK, Keil FC (2015) Searching for explanations: How the Internet inflates estimates of internal knowledge. Journal of Experimental Psychology General 144: 674–687. 10.1037/xge0000070 25822461

[29] Critchley HD, Wiens S, Rotshtein P, Ohman A, Dolan RJ (2004) Neural systems supporting interoceptive awareness. Nat Neurosci 7: 189–195. 10.1038/nn1176 14730305

[30] Craig AD, Chen K, Bandy D, Reiman EM (2000) Thermosensory activation of insular cortex. Nat Neurosci 3: 184–190. 10.1038/72131 10649575

[31] Dronkers NF (1996) A new brain region for coordinating speech articulation. Nature 384: 159–161. 10.1038/384159a0 8906789

[32] Craig AD (2009) How do you feel—now? The anterior insula and human awareness. Nat Rev Neurosci 10: 59–70. 10.1038/nrn2555 19096369

[33] Heining M, Young AW, Ioannou G, Andrew CM, Brammer MJ, et al (2003) Disgusting smells activate human anterior insula and ventral striatum. Ann N Y Acad Sci 1000: 380–384. 14766651 10.1196/annals.1280.035

[34] Zatorre RJ, Fields RD, Johansen-Berg H (2012) Plasticity in gray and white: neuroimaging changes in brain structure during learning. Nat Neurosci 15: 528–536. 10.1038/nn.3045 22426254 PMC3660656

[35] Spence C, Okajima K, Cheok AD, Petit O, Michel C (2015) Eating with our eyes: From visual hunger to digital satiation. Brain Cogn. 10.1016/j.bandc.2015.08.006 26432045

